# The Medium-Chain Fatty Acid Receptor GPR84 Mediates Myeloid Cell Infiltration Promoting Steatohepatitis and Fibrosis

**DOI:** 10.3390/jcm9041140

**Published:** 2020-04-16

**Authors:** Tobias Puengel, Steve De Vos, Jana Hundertmark, Marlene Kohlhepp, Nurdan Guldiken, Philippe Pujuguet, Marielle Auberval, Florence Marsais, Kenji F. Shoji, Laurent Saniere, Christian Trautwein, Tom Luedde, Pavel Strnad, Reginald Brys, Philippe Clément-Lacroix, Frank Tacke

**Affiliations:** 1 Department of Medicine III, RWTH-University Hospital Aachen, 52074 Aachen, Germany; tpuengel@ukaachen.de (T.P.); ngueldiken@ukaachen.de (N.G.); ctrautwein@ukaachen.de (C.T.); tluedde@ukaachen.de (T.L.); pstrnad@ukaachen.de (P.S.); 2 Department of Hepatology & Gastroenterology, Charité University Medicine Berlin, 13353 Berlin, Germany; jana.hundertmark@charite.de (J.H.); marlene.kohlhepp@charite.de (M.K.); 3 Galapagos SA, 102 avenue Gaston Roussel, 93230 Romainville, France; steve.devos@glpg.com (S.D.V.); philippe.pujuguet@glpg.com (P.P.); marielle.auberval@glpg.com (M.A.); florence.marsais@glpg.com (F.M.); kenji.shoji@glpg.com (K.F.S.); laurent.saniere@glpg.com (L.S.); philippe.clement-lacroix@glpg.com (P.C.-L.); 4 Galapagos NV, Generaal De Wittelaan L11 A3, 2800 Mechelen, Belgium; reginald.brys@glpg.com

**Keywords:** monocytes, macrophages, neutrophils, fatty acid, medium-chain fatty acid, G protein-coupled receptor 84 (GPR84), apoptosis signal-regulating kinase 1 (ASK-1), therapy, fibrosis, NASH

## Abstract

Medium-chain fatty acids (MCFAs) have been associated with anti-steatotic effects in hepatocytes. Expression of the MCFA receptor GPR84 (G protein-coupled receptor 84) is induced in immune cells under inflammatory conditions and can promote fibrogenesis. We aimed at deciphering the role of GPR84 in the pathogenesis of non-alcoholic steatohepatitis (NASH), exploring its potential as a therapeutic target. GPR84 expression is upregulated in liver from patients with non-alcoholic fatty liver disease (NAFLD), correlating with the histological degree of inflammation and fibrosis. In mouse and human, activated monocytes and neutrophils upregulate GPR84 expression. Chemotaxis of these myeloid cells by GPR84 stimulation is inhibited by two novel, small molecule GPR84 antagonists. Upon acute liver injury in mice, treatment with GPR84 antagonists significantly reduced the hepatic recruitment of neutrophils, monocytes, and monocyte-derived macrophages (MoMF). We, therefore, evaluated the therapeutic inhibition of GPR84 by these two novel antagonists in comparison to selonsertib, an apoptosis signal-regulating kinase 1 (ASK1) inhibitor, in three NASH mouse models. Pharmacological inhibition of GPR84 significantly reduced macrophage accumulation and ameliorated inflammation and fibrosis, to an extent similar to selonsertib. In conclusion, our findings support that GPR84 mediates myeloid cell infiltration in liver injury and is a promising therapeutic target in steatohepatitis and fibrosis.

## 1. Introduction

Nonalcoholic fatty liver disease (NAFLD) is the most common liver disease worldwide, making NAFLD and its inflammatory subtype, nonalcoholic steatohepatitis (NASH), a major risk factor for the development of cirrhosis and hepatocellular carcinoma [1,2]. As a result of excessive intake of dietary lipids, sugars, and other carbohydrates, NAFLD is characterized by an altered composition of circulating fatty acids, dominated by saturated fatty acids and permanently elevated levels of blood sugar [3]. Upon disease progression, hepatic fibrosis is the key characteristic determining liver-related and overall mortality in NAFLD [4]. Altered dietary intake of lipids and carbohydrates strongly affects the liver—the primary metabolic organ. High levels of blood sugar and accumulation of saturated fatty acids cause lipotoxicity, leading to the activation of immune pathways and causing cell death, as a signal of hepatic stress response [1,5]. Pro-inflammatory signals like chemokines and cytokines, as well as danger-associated molecular patterns (DAMPs) or pathogen-associated molecular patterns (PAMPs) are involved in the recruitment of innate immune cells [6]. During acute or chronic liver injury, infiltrating immune cells from the blood circulation, such as monocytes or neutrophils, and tissue resident non-parenchymal cells like Kupffer cells are the key regulators orchestrating the progression of NAFLD and fibrosis [7,8]. These myeloid cells acquire a particular inflammatory phenotype in steatohepatitis, which is (partly) mediated by fatty acids [9]. While free fatty acids are generally considered harmful, the specific role of medium-chain fatty acids (MCFA) in NAFLD and NASH is not fully understood. Using a human hepatocyte-like cell line (LO2) exposed to steatogenic stimuli, the long-chain fatty acid palmitate, but not MCFA, induced apoptosis, oxidative stress, and inflammation [10]. Moreover, MCFA was found to reduce lipid accumulation by regulating key lipid-sensing genes in this in vitro model [10].

GPR84 (G protein-coupled receptor 84) is a receptor for MCFA, which is induced in various immune cells under inflammatory conditions [11]. MCFA, as well as other metabolites, are nowadays considered to be important regulators of inflammatory and metabolic responses [12]. In a dietary model of obesity, GPR84-deficient mice developed reduced hepatic steatosis compared to wildtype mice, upon exposure to long-chain fatty acids [13]. In addition, GPR84 was suggested to promote inflammatory activation and phagocytosis of human and murine macrophages [11]. Importantly, GPR84-deficient mice developed reduced fibrosis in a model of kidney fibrosis (adenine-induced chronic kidney disease), although the exact mechanism(s) remained elusive [14].

In this study, we set out experiments to decipher the role of GPR84 in the pathogenesis of NAFLD and to explore the potential of therapeutically targeting GPR84 against NASH and liver fibrosis. Herein, we demonstrate that GPR84 expression is upregulated upon NASH induction in mice and men. GPR84 inhibition, achieved with two novel, structurally distinct small molecule antagonists (CpdA and CpdB) in mouse models of acute and chronic liver injury, was shown to reduce hepatic recruitment of neutrophils, monocytes, and monocyte-derived macrophages, ultimately ameliorating hepatic inflammation and fibrosis. These data indicate a role of MCFA for myeloid cell recruitment during NASH progression and support the clinical development of GPR84 antagonists in NAFLD.

## 2. Material and Methods

### 2.1. Human Liver Samples

Liver biopsies of NAFLD patients (*n* = 19) were collected and processed, as described previously [15]. Healthy controls (*n* = 5) were defined from subjects, who underwent liver biopsy because of mildly elevated transaminase levels (levels lesser than twice the upper limit of normal) and were deemed normal upon histological analysis. All liver sections were graded and staged by an experienced and blinded pathologist, according to the NAFLD activity score (NAS) [16]. A written informed consent was initially obtained from all patients and the analysis was in compliance with the 1975 Declaration of Helsinki, as reflected in an approval by the Human Subjects Committees of the participating centers.

### 2.2. Animal Experiments

C57BL6/J wildtype (WT) male mice were purchased from Janvier Labs, France, at the age of 6 weeks, and housed at the Animal Facility of Galapagos SA, Romainville, France. After 2 weeks of acclimation, the animals were randomized into the different treatment groups. All in vivo experiments were performed with mice at 9 to 18 weeks of age. All in vivo experiments were carried out in a dedicated pathogen-free facility (22 °C). Animal care was in accordance with the French guidelines covering the use of animals for scientific research. All procedures involving animals, including housing and care, method of euthanasia, and experimental protocols, were conducted in accordance with a code of practice established by the local ethics committee (Galapagos). Experiments were conducted under the establishment license number C-93-063-06, conforming to the Animal Research: Reporting of In Vivo Experiments (ARRIVE) guidelines.

### 2.3. Pharmacological Agents and Administration

Compound A (CpdA) and Compound B (CpdB) (described in patent numbers WO2016169911 and WO2014095798) were profiled in a diverse set of selectivity assays. CpdA and Cpd B were fully selective over close homologs GPR43 (FFA2R) and GPR41 (FFA3R) (no inhibition up to 10 µM in [Ca^2+^] i flux assay), and > 100-fold selective over 123 and other G protein-coupled receptors (GPCR)s on a Millipore GPCR Profiler panel. CpdA and Cpd B were remarkably selective over a kinase panel—Cpd B displayed no inhibitory activity out of a selection of 152 kinases (Reaction Biology, Malvern, PA, USA), while CpdA only weakly inhibited maternal embryonic leucine zipper kinase (MELK; 57% inhibition at a concentration of 10 µM). This inhibitory activity was not considered to be relevant with regards to the dose used in animal models. Similar high selectivity was also observed against a diverse panel of 67 enzymes, ion channels, and transporters (Table 1). CpdA and CpdB were dissolved/suspended in water containing methylcellulose 0.5% + 1 eq HCl (VWR, France) and administered at 30 mg/kg q.d. Selonsertib (synthetized by Galapagos’ chemists (batch 6) at a purity of 95%) was dissolved/suspended in water containing methylcellulose 0.5% + 1 eq HCl (VWR, France) and administered at 15 mg/kg b.i.d. The solutions/suspensions were kept at room temperature, in dark, under constant magnetic stirring. The volume administered was 10 mL/kg. Volume was adapted according to the weight of the mice (mean of the group), once per week.

### 2.4. Induction of Liver Injury and Pharmacological Treatment

Chronic liver injury was induced either by feeding a methionine-choline deficient (MCD) diet (Research Diet, New Brunswick, USA no. D12451) for 8 weeks, or by feeding a choline-deficient, L-amino acid-defined, high-fat diet (CDAHFD) (Research Diet, New Brunswick, USA, Ref A06071302 containing 60 kcal% of fat and 0.1% of methionine) for 10 weeks, and by carbon tetrachloride (CCl_4_) (Merck, Darmstadt, Germany) dissolved in corn oil, injected intraperitoneally (IP) at 0.6 mL/kg body weight (BW), twice per week for 8 weeks. Overall, three different compounds were tested in the in vivo studies, two small molecule GPR84 antagonists (CpdA and CpdB) and, as a positive control comparator, selonsertib, a ASK1-inhibitor under clinical investigation [17]. Based on a phase II clinical trial and several experimental studies in rodents, it was assumed that selonsertib acts on hepatocytes (reducing cell death), stellate cells (reducing fibrogenic activation), and on macrophages (reducing inflammatory activation), because ASK1 was found to be activated in all these cell types. The compounds were dispensed in sterile water mixed with 0.5% methylcellulose. In chronic CCl_4_ and MCD-diet induced liver injury, mice received GPR84 antagonists via oral gavage once daily, at a concentration of 30 mg/kg BW, selonsertib via oral gavage twice daily, at a concentration of 15 mg/kg BW or an equal amount of vehicle, all from the beginning of week 5 onwards. Accordingly, in the acute liver injury model, liver mice received one intraperitoneal application of CCl_4_ at 0.6 mL/kg BW, and received the compounds two (GPR84 antagonists) or three times (selonsertib) at 0 h, 12 h, and 24 h, respectively, and the results were assessed after a total period of 36 h.

### 2.5. Phenotypic Assessment and Model Endpoints

Hematoxylin-eosin (H&E) and Sirius red staining of the mouse liver samples were conducted according to the established protocols. Sirius red area fractions were analyzed using ImageJ (public domain). Colorimetric tests for hydroxyproline and triglyceride liver content were conducted as described [18]. The NAFLD activity score was assessed by an external expert histology service provider (Histalim) blinded to the treatment group. Immunohistochemistry for F4/80 (Abcam, UK) was performed according to the standard protocols on paraffin-embedded liver sections. Hepatic leukocytes were analyzed by multi-color flow cytometry, using a BD FACSCanto™ II system (BD Biosciences, Le Pont de Claix, France) as previously described [18]. GPR84 gene expression was measured from total RNA in liver MoMF and Kupffer cells, after isolation, through fluorescent-activated cell sorting, using an Aria-II (BD Biosciences, Heidelberg, Germany) of C57BL/6 WT mice at 4 weeks of MCD diet, as previously described [18].

### 2.6. Stimulation and Chemotaxis of Primary Human Monocytes, Macrophages and Neutrophils

All cells were harvested from healthy volunteers. Human neutrophils were isolated from buffy coats based on a procedure of gradient density centrifugation. Human primary CD14^+^ monocytes were isolated from buffy coats using the MACS technology and were differentiated to monocyte-derived macrophages by stimulation with 100 ng/mL M-CSF for 10 days. Human differentiated macrophages and isolated human neutrophils were stimulated with a pro-inflammatory trigger, lipopolysaccharide (LPS at 10 ng/mL for macrophages or 200 ng/mL for neutrophils), or with an M2 polarizing trigger, interleukin-4 (IL-4, 10 ng/mL). Cells were harvested and lysed for mRNA isolation at different time points, post stimulation. Gene expression was assessed by quantitative real-time polymerase chain reaction (qPCR). Primer sequences are available upon request. Ligand induced chemotaxis of human neutrophils towards GPR84-specific agonists (Embelin at 10 µM or sodium decanoate (capric acid) at 0.5 mM) was analysed as functional cellular activity.

### 2.7. Chemotaxis Assays for Murine Bone-Marrow Monocytes and Neutrophils

Bone marrow of the untreated WT mice at 8 weeks of age was used to isolate the immune cells for transwell migration assays. A single cell suspension was achieved by flushing cold RPMI-1640 through the bones, and grinding the received bone marrow through a 70-µm cell strainer. After lysis of red blood cells by Pharmlyse (BD Biosciences, San Jose, CA, USA), 1 × 10^6^ cells in RPMI-1640 were placed in the upper compartment of the 5-µm transwell migration chamber [19]. Migration was analyzed towards stimulation with a 3-µM embelin, a surrogate GPR84 agonist [20], or a 100-µM myristic acid (an MCFA) dissolved in combination with bovine serum albumin (BSA) in the lower compartment. The chemotactic cytokines, 5 nM CCL2 or 1 nM CXCL1, were used as positive controls for monocyte or neutrophil migration, respectively. After 2 h of incubation at 37 °C, the cells in the lower compartment were analyzed by flow cytometry [19].

### 2.8. In Situ Hybridization

In situ hybridization was performed to localize GPR84 mRNA expression in formalin-fixed, paraffin-embedded mouse liver sections, using the RNAscope 2.5 LSx reagent kit red (Advanced Cell Diagnostics, Bio-Techne, France). Briefly, paraffin-embedded liver sections were cut at 4 µm, air dried overnight, baked at 60 °C for 1 h, dewaxed, and air dried before pretreatments. The standard pretreatment protocol was used according to the manufacturer’s instructions. RNAscope probes for GPR84 (Mm-Gpr84; catalog number 438378) and, respectively, positive and negative control probes as UBC (Mm-Ubc; catalog number 310778) and dapB (catalog number 312038) were used. Detection of probe binding was performed using the RNAscope Detection Kit, based on alkaline phosphatase detection with Fast Red chromogen. Finally, the sections were counterstained with hematoxylin.

### 2.9. Statistics

All experimental data from mice are presented as mean ± standard deviation (SD). Differences between groups were evaluated by two-tailed unpaired Student *t*-test, one-way ANOVA with post-hoc testing and Spearman’s linear correlation analysis (GraphPad Prism, GraphPad Software Inc., La Jolla, CA, USA).

## 3. Results

### 3.1. Inflammatory Induction of GPR84 in Mice and Men Promotes Myeloid Cell Chemotaxis

**Figure 1:**
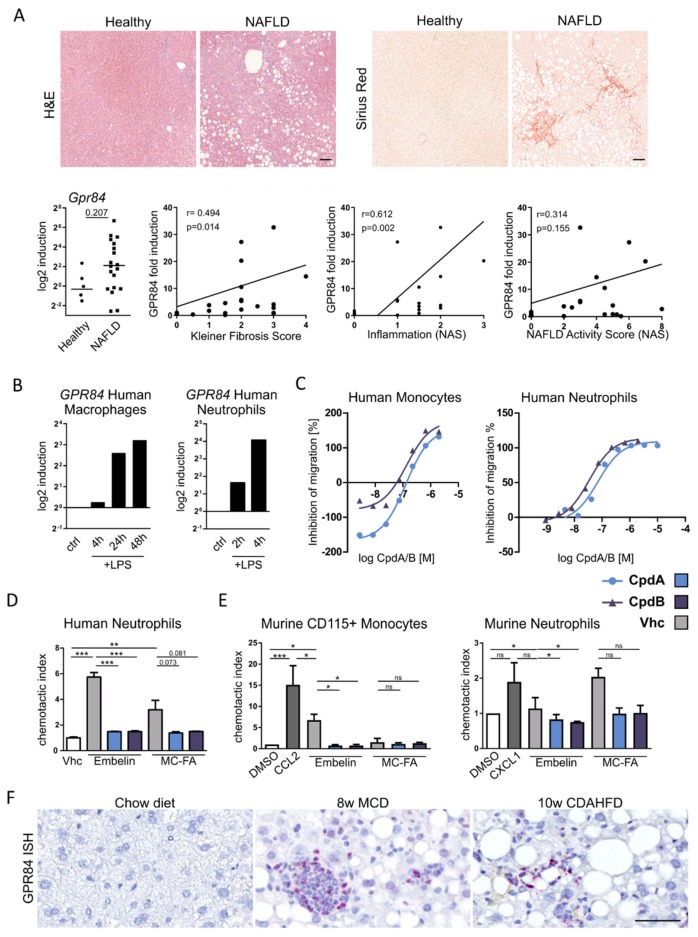
**Inflammatory induction of GPR84 in mice and men promotes myeloid cell chemotaxis in vitro.** (**A**) Liver biopsies of healthy control (*n* = 5) and NAFLD patients (*n* = 19) were analyzed. Representative H&E and Sirius Red stainings of paraffin-embedded liver sections revealed steatosis and inflammation in NAFLD patients. GPR84 gene expression tended to be elevated in livers of NAFLD patients compared to healthy controls and was significantly correlated with the histological degree of inflammation and fibrosis. (**B**) GPR84 expression of primary human macrophages and neutrophils upon LPS stimulation in vitro. Representative data from one human donor. (**C**) Dose-dependent inhibition with two GPR84 antagonists on embelin-induced migration of human primary monocytes and neutrophils. (**D**) Migration of human neutrophils was induced by embelin (GPR84 agonist) or medium-chain fatty acids (MCFA) and was effectively inhibited by the two GPR84 antagonists. (**E**) Migration of CD115+ monocytes and neutrophils from mouse bone marrow towards embelin or MCFAs. (**F**) GPR84 mRNA in situ hybdridization in representative liver sections of experimental dietary mouse models. Scale bar, 100 µm. Data are presented as mean ± SD. * *p* < 0.05, ** *p* < 0.01, *** *p* < 0.001 (unpaired Student’s *t*-test in A, ANOVA in D + E, Spearman’s r, and *p*-values of linear correlation analysis in A). Abbreviations: CCL2, C-C motif chemokine 2; CpdA/B, compound A/B; LPS, lipopolysaccharide; Vhc, vehicle; MCD diet, methionine choline deficient diet; and CDAHFD, choline-deficient, L-amino acid-defined, high-fat diet.

The MCFA receptor GPR84 is highly expressed in leukocytes, particularly neutrophils and macrophages, and is associated with the inflammatory activation of these myeloid immune cells [21]. In order to explore the potential relevance of this receptor in metabolic liver disease, we analyzed liver biopsies of NAFLD patients (*n* = 19) and compared the results to healthy controls (*n* = 5) (Table 2). GPR84 tended to be increased in NAFLD compared to healthy livers (not significant, Figure 1A). In diseased livers, GPR84 gene expression was associated with the inflammation grading of the NAFLD Activity Score (NAS), as well as with the fibrosis stage, but not with steatosis (Figure 1A). In line, the stimulation of primary human macrophages or neutrophils with LPS resulted in increased expression of *GPR84* (Figure 1B), while stimulation of the human macrophages with IL-4 decreased *GPR84* expression in vitro.

GPR84 expression has been linked to various inflammatory properties of myeloid immune cells, including chemotaxis [22]. This response was used to test the efficacy of two novel, small molecule antagonists of GPR84 (CpdA and CpdB, described in patent numbers WO2016169911 and WO2014095798), belonging to two different chemical series. These compounds specifically inhibit GPR84 activation in cell membranes stably overexpressing human GPR84 (see Table 1). Neither CpdA nor CpdB inhibit the related free fatty acid (FFA) receptors FFA1, FFA2, and FFA3. However, both compounds effectively inhibited the embelin-induced migration of human primary monocytes (EC_50_ = 120 nM for CpdA, EC_50_ = 118 nM for CpdB) and human neutrophils (EC_50_ = 79 nM for CpdA, EC_50_ = 35 nM for CpdB) in vitro, at nanomolar concentrations, in a dose-dependent manner (Figure 1C). Moreover, human neutrophil migration was strongly induced by the GPR84 agonist embelin (5 µM), as well as by MCFA (0.5 mM), and both antagonist compounds blocked this migration response to the ligands at an effective concentration of 10 µM in vitro (Figure 1D).

As we aimed at exploring the therapeutic potential of GPR84 inhibition in mouse models of steatohepatitis, we next subjected mouse myeloid cells from bone marrow to transwell chemotaxis assays, with the chemokines CCL2 and CXCL1 as positive controls for monocyte or neutrophil migration, respectively (Figure 1E). In vitro, neutrophils were attracted by embelin as well as MCFA, whereas monocyte migration was stimulated by embelin, but not significantly by MCFA (at the dose and time-point investigated). These experiments indicated that mouse monocyte and neutrophil migration could be effectively reduced by the two GPR84 antagonists in vitro (Figure 1E). Importantly, in situ hybridization was performed to localize GPR84 mRNA expression in control and experimental dietary models in mice, using the methionine choline deficient (MCD) diet and choline-deficient, L-amino acid-defined, high-fat diet (CDAHFD). In situ hybdridization indicated higher GPR84 expression in periportal areas of NASH mouse livers, where the inflammatory cells accumulate. Collectively, our findings demonstrated that GPR84 is upregulated in conditions of human and murine NAFLD. GPR84 is induced in myeloid immune cells upon inflammation, and two small molecule GPR84 antagonists (CpdA and CpdB) can pharmacologically block embelin or MCFA-dependent migration of monocytes and neutrophils.

### 3.2. Pharmacological Antagonism of GPR84 Inhibits the Infiltration of Hepatic Neutrophils and Macrophages in Acute Liver Injury

**Figure 2:**
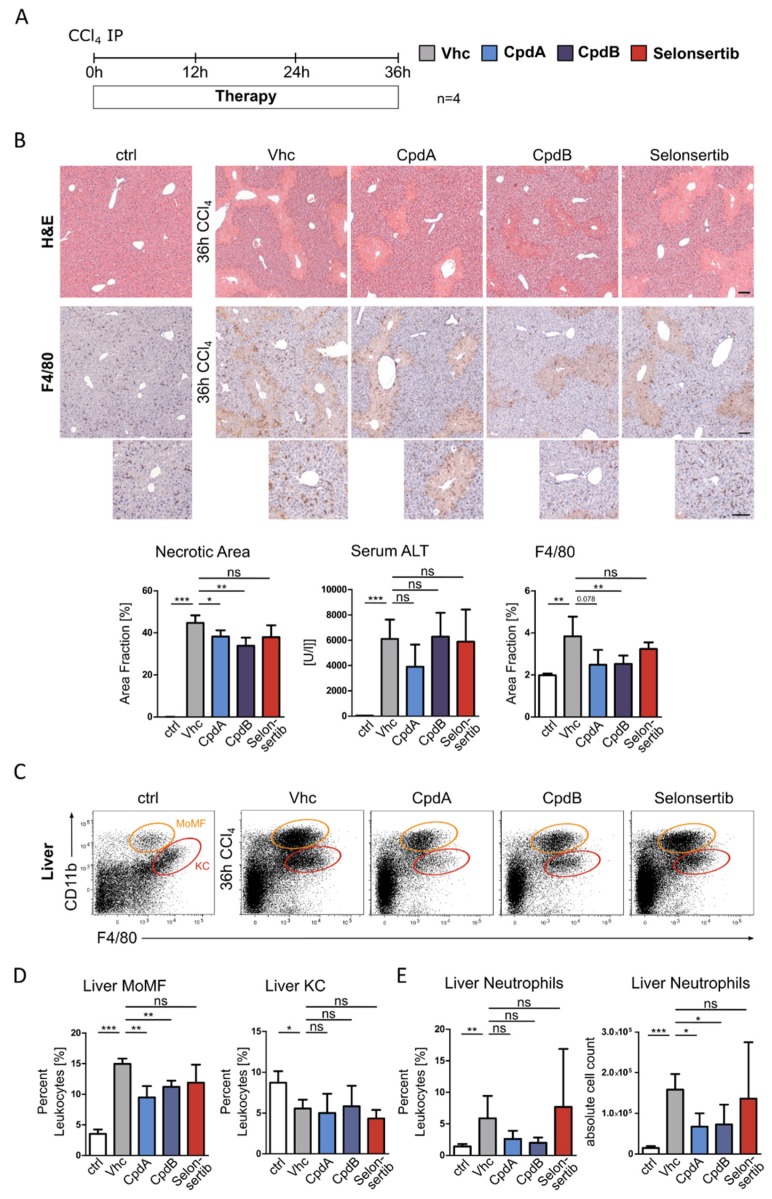
Pharmacological inhibition of GPR84 decreases infiltration of neutrophils and macrophages in CCl_4_-induced acute liver injury. (**A**) In vivo effects of GPR84 inhibition on leukocyte infiltration were assessed in c57bl/6 wildtype mice (*n* = 4), 36 h after being challenged with a single intraperitoneal (IP) injection of carbon tetrachloride (CCl_4_). Mice were treated by oral gavage with different GPR84 antagonists (30 mg/kg BW, at 0 h, and 24 h) or selonsertib (15 mg/kg BW, at 0 h, 12 h, and 24 h). (**B**) Liver histology (H&E staining), immunohistochemistry for the macrophage marker F4/80 (overview and higher magnification of periportal areas below) and serum alanine transaminase (ALT) levels. (**C**–**E**) Immune cell infiltration was characterized from mouse livers by FACS (representative FACS plots shown in C), revealing reduced numbers of hepatic neutrophils (E, relative and absolute counts are shown), as well as monocyte derived macrophages (MoMF), after a CCl_4_ injection, in case of GPR84 inhibition, while the Kupffer cells (KC) remained unchanged (**D**). All images were taken at ×10 objective magnification; scale bar, 100 µm. Data are presented as mean ± SD, based on *n* = 4 per group. * *p* < 0.05, ** *p* < 0.01, *** *p* < 0.001 (one-way ANOVA). Abbreviations are: CpdA/B, compound A/B; KC, Kupffer cells; MoMF, monocyte-derived macrophages; and Vhc, vehicle.

To translate our in vitro findings into an in vivo setup we assessed the effects of GPR84 inhibition on leukocyte infiltration into the liver, employing the acute carbon tetrachloride (CCl_4_) model. Parallel to induction of acute liver injury by a single intraperitoneal (IP) injection of CCl_4_, mice received either one of the GPR84 antagonists, vehicle or selonsertib (an active control for the subsequent fibrosis experiments) by oral gavage (Figure 2A). While we observed a mild reduction in the extent of acute liver injury, assessed by necrotic areas on histology and serum alanine transaminase (ALT) levels, in mice treated with the GPR84 antagonists, pharmacological GPR84 inhibition reduced the massive accumulation of macrophages induced by CCl_4_ injury, as observed by immunohistochemistry for the macrophage marker F4/80 (Figure 2B). The ASK1 inhibitor selonsertib neither significantly modified hepatic necrosis nor F4/80+ cell infiltration (Figure 2B). The infiltration of hepatic immune cells was further characterized by flow cytometry, allowing to distinguish liver-resident Kupffer cells (KC) from monocyte-derived macrophages (MoMF) in injured livers (Figure 2C). GPR84 antagonism, but not selonsertib, significantly reduced MoMF and neutrophil infiltration, following acute liver injury in mice, while leaving the KC unaffected (Figure 2D–E). These data supported the pharmacological inhibition of GPR84 as an effective strategy to reduce recruitment of myeloid cells to injured liver.

### 3.3. Reduced Hepatic Neutrophil and Macrophage Accumulation by GPR84 Inhibition Ameliorates Steatohepatitis and Fibrosis in Dietary Models of Fatty Liver Disease

In mice and men, infiltration of inflammatory myeloid immune cells into chronically injured liver is closely associated with the progression of hepatic inflammation and fibrosis [6]. The ideal dietary model reflecting the human disease does not exist. One of the most commonly employed model is that of the methionine choline deficient (MCD), which reflects oxidative stress in steatotic hepatocytes, programmed cell death, and continuous disease progression with moderate fibrosis [23]. In contrast to the MCD model, choline-deficient, L-amino acid-defined, high-fat diet (CDAHFD)-fed mice also develop steatohepatitis and fibrosis, but without the reduction in bodyweight that is typical for MCD [24]. We therefore investigated the effects of therapeutically targeting GPR84 during steatohepatitis and fibrosis progression, in these dietary models.

**Figure 3:**
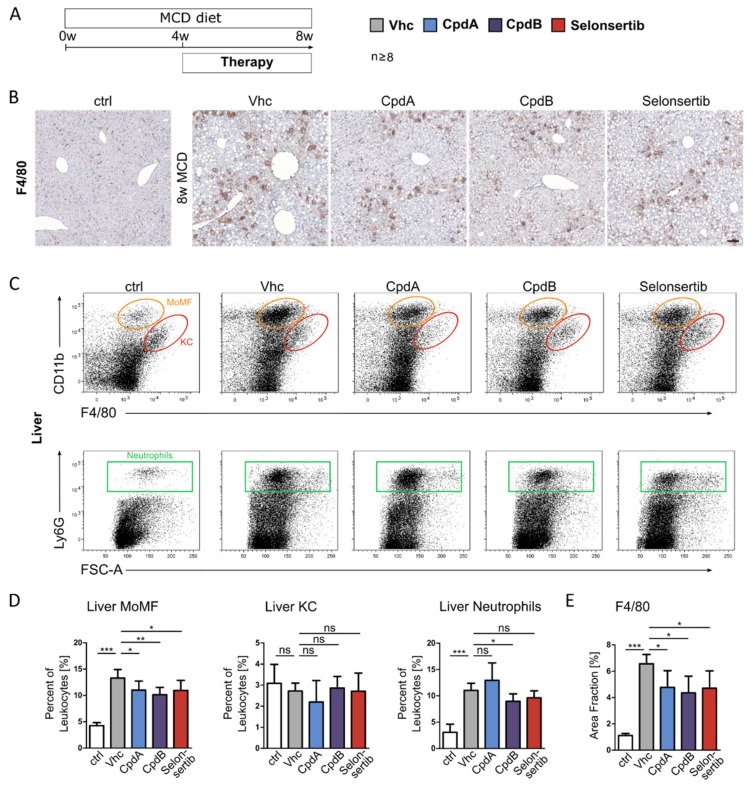
**GPR84 inhibition reduces hepatic neutrophil and macrophage accumulation in MCD diet-induced steatohepatitis.** (**A**) Mice (*n* ≥ 8) were fed with a methionine choline deficient (MCD) diet for a period of 8 weeks. Oral pharmacological therapy was conducted over the last 4 weeks of liver injury induction with the two GPR84 antagonists (30 mg/kg BW, PO q.d.) and selonsertib (15 mg/kg BW, PO b.i.d.) as a positive control. (**B**) Representative pictures of immunohistochemistry staining for the pan-macrophage marker F4/80. (**C**) Representative FACS plots showing CD11b+ F4/80+ hepatic monocyte-derived macrophages (MoMF, orange circle), CD11b+ F4/80++ Kupffer cells (KC, red circle), or Ly6G+ neutrophils (green rectangle). (**D**,**E**) While the effects on hepatic neutrophils were less pronounced, GPR84 or ASK1 inhibition significantly reduced MoMF numbers (by FACS, D) and F4/80 macrophages (by immunohistochemistry, E). All images were taken at ×10 magnification; scale bar, 100 µm. Data are presented as mean ± SD based on *n* ≥ 8 per group. * *p* < 0.05, ** *p* < 0.01, *** *p* < 0.001 (one-way ANOVA). Abbreviations: CpdA/B, compound A/B; and Vhc, vehicle.

Mice were fed the MCD diet for a total period of 8 weeks, and pharmacological therapy was conducted over the last 4 weeks of liver injury induction with the two GPR84 antagonists, and the ASK1 inhibitor selonsertib as a positive control (Figure 3A). In the chronic MCD diet model, GPR84 or ASK1 inhibition significantly reduced CD11b+ F4/80+ MoMFs (orange circle), as assessed by immunohistochemistry staining for the pan-macrophage marker F4/80 and FACS analysis, while the effects on hepatic Ly6G+ neutrophils (green rectangle) were less pronounced (Figure 3B–E). CD11b+ F4/80++ Kupffer cells (KC, red circle) as well as liver lymphocytes (Figure S1: Immune cells in MCD diet induced steatohepatitis and fibrosis) were not affected upon pharmacological treatment (Figure 3C–D).

**Figure 4:**
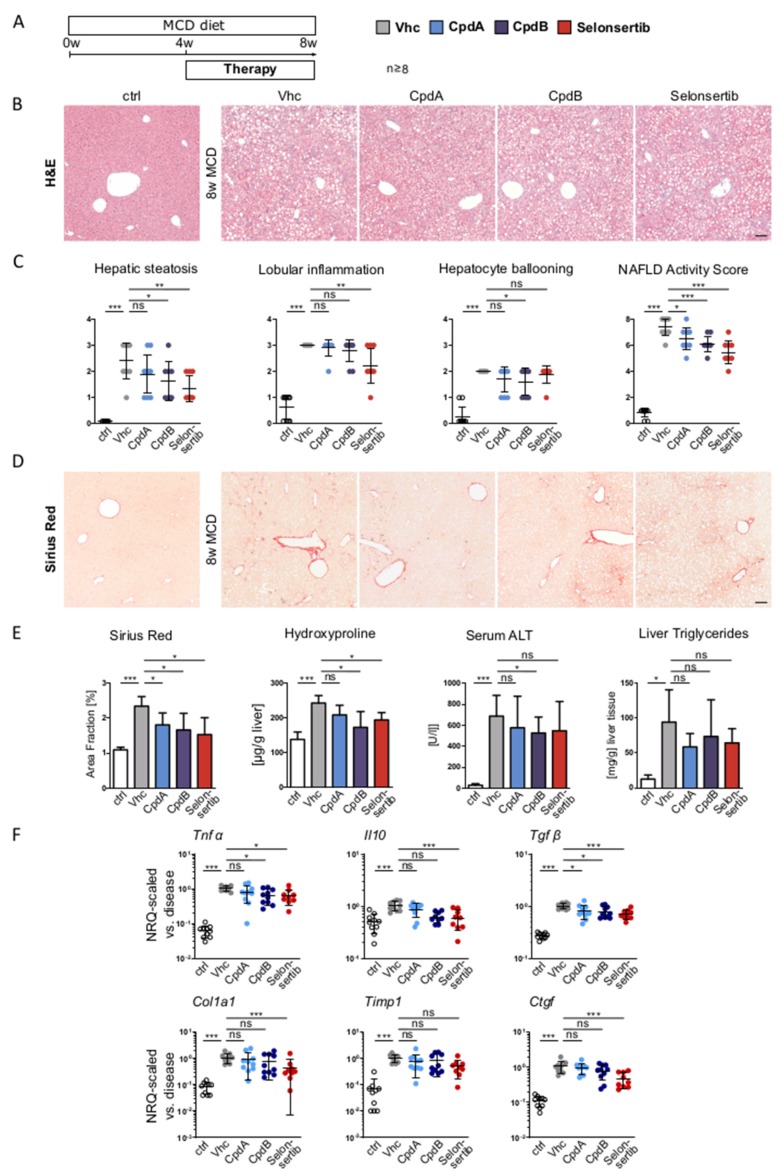
**Pharmacologic inhibition of GPR84 ameliorates steatohepatitis and fibrosis in MCD diet-induced fatty liver disease.** (**A**) Mice (*n* ≥ 8) were fed with a methionine choline deficient (MCD) diet for a period of 8 weeks. Oral pharmacological therapy was conducted over the last 4 weeks of liver injury induction with the two GPR84 antagonists (30 mg/kg BW, PO q.d.) and Selonsertib (15 mg/kg BW, PO b.i.d.) as a positive control. (**B**) Liver histology was evaluated based on H&E stainings, showing severe fibrosing steatohepatitis in MCD fed animals. (**C**) The NAFLD activity score (NAS) was assessed by an external expert pathologist (Histalim), revealing a significantly reduced NAS upon GPR84 or ASK1 inhibition. (**D**) Liver fibrosis was assessed by Sirius-Red-stained liver sections (red: extracellular matrix deposition). (**E**) Quantification of liver fibrosis by Sirius-Red-stained areas and hepatic hydroxyproline content, liver injury by serum ALT and steatosis by hepatic triglyceride concentrations. (**F**) Inflammation- and fibrosis-related gene expression in total liver measured by RT-qPCR. All images were taken at ×10 magnification; scale bar, 100 µm. Data are presented as mean ± SD based on *n* ≥ 8 per group. * *p* < 0.05, ** *p* < 0.01, *** *p* < 0.001 (one-way ANOVA). Abbreviations: CpdA/B, compound A/B; Vhc, vehicle; Tnfα, tumor necrosis factor alpha; Il10, interleukin 10; Tgfβ, transforming growth factor beta; Col1a1, collagen type I, alpha 1; Timp1, tissue inhibitor of metalloproteinases 1; and Ctgf, connective tissue growth factor.

From the same experiment (Figure 4A), liver histology was evaluated based on H&E staining, showing severe fibrotic steatohepatitis in MCD diet-fed animals (Figure 4B). Histological scoring by an independent and blinded expert pathologist (Histalim) revealed variably ameliorated hepatic steatosis, ballooning, and lobular inflammation, upon pharmacological inhibition of GPR84 and ASK1, resulting in a significantly reduced NAFLD Activity Score (NAS), overall (Figure 4C). Importantly, liver fibrosis was reduced in the GPR84 antagonist- or selonsertib-treated groups, as assessed by quantification of Sirius-Red-stained liver sections and hepatic hydroxyproline content (Figure 4D–E). Serum ALT levels and hepatic triglyceride concentrations did not show a significant reduction upon pharmacological treatment (Figure 4D–E). Reduction of hepatic neutrophils and macrophages as well as reduced liver fibrosis was accompanied by a reduction of gene expression levels associated with inflammation (*Tnfα*, *Il10*, and *Tgfβ*) and less pronounced fibrosis (*Col1a1*, *Timp*, and *Ctgf*) (Figure 4F).

**Figure 5:**
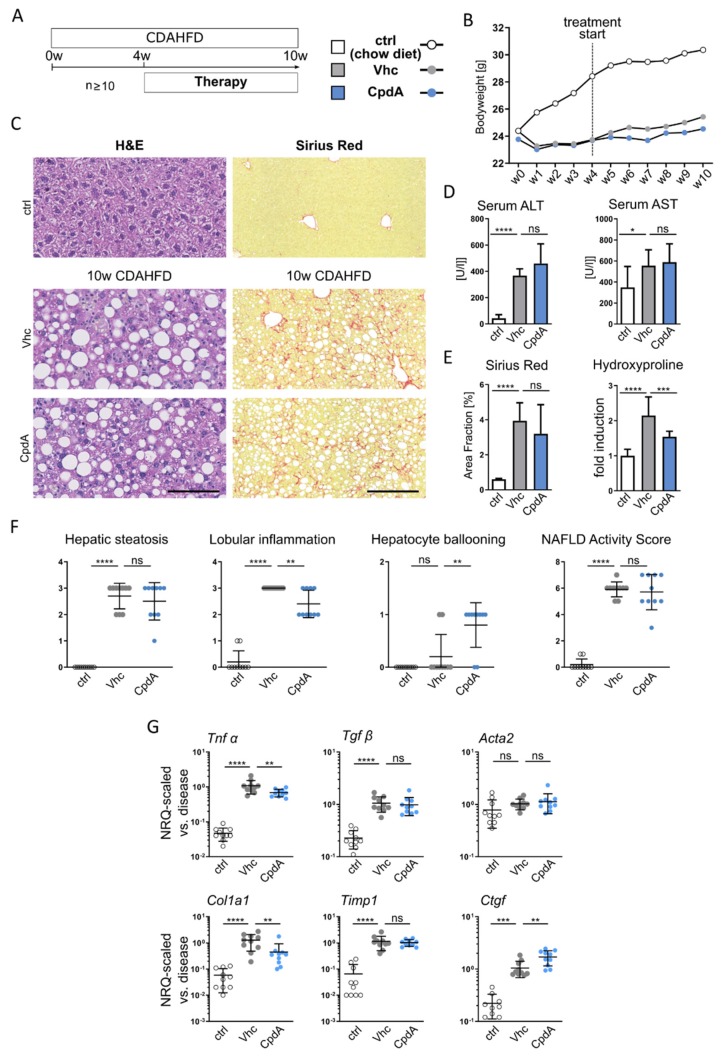
**Pharmacologic inhibition of GPR84 ameliorates inflammation and fibrosis in CDAHFD induced NASH.** (**A**) Mice were fed with a choline-deficient, L-amino acid-defined, high-fat diet (CDAHFD) for a period of 10 weeks. Oral pharmacological therapy was conducted over the last 6 weeks of liver injury induction with the GPR84 (CpdA) antagonist (30 mg/kg BW, PO q.d.). (**B**) Development of bodyweight over time. (**C**–**E**) Representative H&E and Sirius Red stainings showing steatohepatitis and fibrosis in CDAHFD-induced NASH. Assessment of liver injury by serum alanine transaminase (ALT) and aspartate transaminase (AST), and liver fibrosis quantification by Sirius-Red-stained areas and fold induction of hepatic hydroxyproline content (**F**) The NAFLD activity score (NAS) was conducted by an external expert pathologist (Histalim), indicating a significant reduction of lobular inflammation upon GPR84 inhibitor treatment. (**G**) RT-qPCR from total liver inflammation- and fibrosis-related gene expression levels. All images were taken at ×10 objective magnification; scale bar, 100 µm. Data are presented as mean ± SD based on *n* ≥ 8 per group. * *p* < 0.05, ** *p* < 0.01, *** *p* < 0.001 (one-way ANOVA). Abbreviations: CpdA, compound A; Vhc, vehicle; Tnfα, tumor necrosis factor alpha; Tgfβ, transforming growth factor beta; Acta2, alpha-actin-2; Col1a1, collagen type I, alpha 1; Timp1, tissue inhibitor of metalloproteinases 1; and Ctgf, connective tissue growth factor.

Pharmacological treatment in the CDAHFD model was conducted over the last 6 weeks of the 10-weeks diet administration, with one of the GPR84 antagonists (CpdA) (Figure 5A). In the chronic CDAHFD model, mice largely maintained their bodyweight over time, while control chow-fed animals continuously gained weight (Figure 5B). Liver histology revealed steatohepatitis und relevant fibrosis. While serum ALT and AST levels were unaffected, quantification of the Sirius Red area fraction and hepatic hydroxyproline content revealed a significant reduction of liver fibrosis, upon pharmacological treatment with the GPR84 antagonist (CpdA) (Figure 5C–E). The histological NAS assessment showed a trend towards reduced levels upon pharmacological treatment. In contrast to the MCD diet reduction of NAS, the CDAHFD-fed mice was mainly based on a striking reduction of lobular inflammation, while steatosis was unaffected (Figure 5F). Accordingly, inflammation- (*Tnfα* and *Tgfβ*) and fibrosis- (*Acta2, Col1a1*, *Timp1*, and *Ctgf*) related gene expression levels were reduced in the GPR84 inhibitor treated groups (Figure 5G).

Thus, pharmacological inhibition of GPR84 led to significantly reduced levels of experimental steatohepatitis and liver fibrosis in vivo, to a level comparable to the ASK1 inhibitor selonsertib. Interestingly, therapeutic GPR84 inhibition in both NASH models demonstrated major effects on inflammation and fibrosis, while the effects on steatosis appeared minor. These data prompted us to investigate the pharmacological effects of GPR84 inhibition in the inflammatory driven chronic CCl_4_ model of liver fibrosis.

### 3.4. Therapeutic Inhibition of GPR84 Reduces Macrophage Accumulation and Hepatic Fibrosis in CCl_4_ Induced Chronic Liver Injury

**Figure 6:**
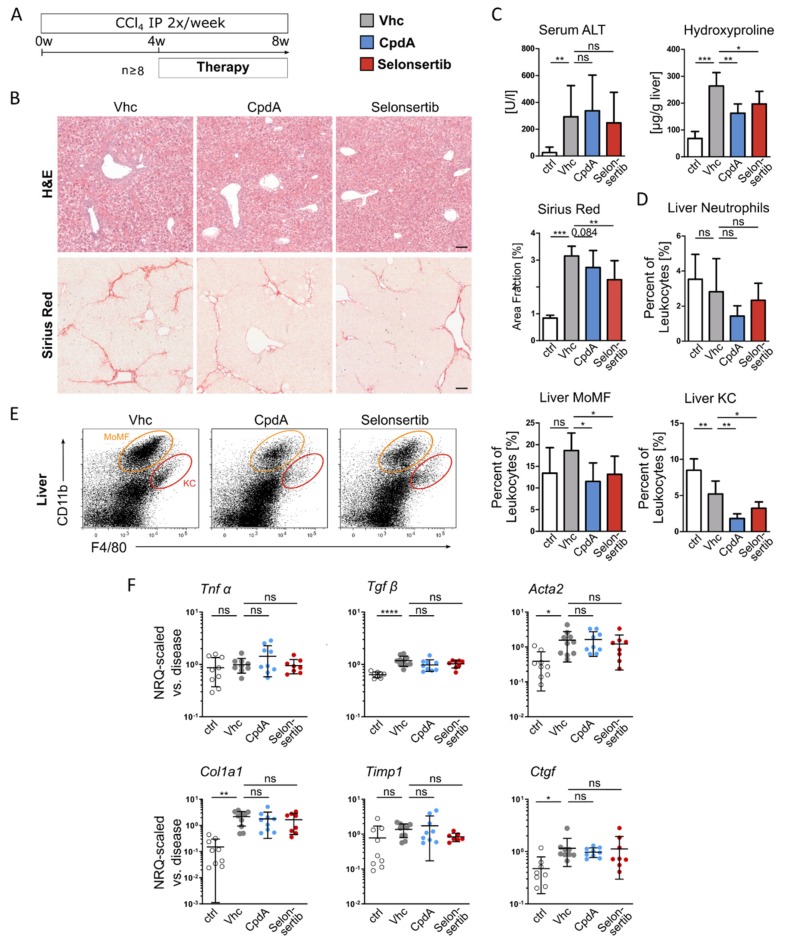
Impaired macrophage recruitment and reduced hepatic fibrosis by therapeutically antagonizing GPR84 in CCl_4_-induced liver fibrosis. (**A**) Liver fibrosis was induced by repetitive IP injections of CCl_4_ (twice per week) for a period of 8 weeks (*n* ≥ 8). GPR84 antagonist (CpdA, 30 mg/kg BW q.d.) and Selonsertib (15 mg/kg BW b.i.d.) were administered by oral gavage over the last 4 weeks of chronic injury induction. (**B**) Hepatic liver injury and fibrosis were assessed by H&E and Sirius-Red staining. (**C**) Quantification of Sirius-Red-positive area fraction, hepatic hydroxyproline content, and ALT serum levels. (**D**,**E**) Analysis of immune cell infiltration by FACS, revealing reduced macrophage numbers upon pharmacological inhibition of GPR84 or ASK1. (**F**) Expression levels of genes related to inflammation and fibrosis by RT-qPCR in total liver. All images were taken at ×10 objective magnification; scale bar, 100 µm. Data are presented as mean ± SD based on *n* ≥ 8 per group. * *p* < 0.05, ** *p* < 0.01, *** *p* < 0.001 (one-way ANOVA). Abbreviations: CpdA/B, compound A/B; KC, Kupffer cells; MoMF, monocyte-derived macrophages; Vhc, vehicle; Tnfα, tumor necrosis factor alpha; Tgfβ, transforming growth factor beta; Acta2, alpha-actin-2; Col1a1, collagen type I, alpha 1; Timp1, tissue inhibitor of metalloproteinases 1; and Ctgf, connective tissue growth factor.

Fibrosis is considered the hallmark feature of NAFLD that is related to disease progression and development of clinical endpoints [25]. Based on our beneficial findings on inflammation and fibrosis in MCD diet and the CDAHFD-fed mice, we tested the antifibrotic effects of GPR84 antagonism in a third experimental model of liver fibrosis, based on repetitive intraperitoneal injections of CCl_4_, twice per week for a period of 8 weeks. Again, the GPR84 antagonist (CpdA) or selonsertib were administered over the last 4 weeks of chronic injury induction (Figure 6A). While the toxic injury in the CCl_4_ model was unaffected according to histology or ALT levels, liver fibrosis was markedly reduced upon GPR84 or ASK1 inhibition, based on Sirius-Red-staining (* *p* = 0.084 upon GPR84 treatment) and hepatic hydroxyproline content (Figure 6B–C). Similar to the MCD diet model, pharmacological GPR84 (and also ASK1) inhibition was associated with lower numbers of hepatic macrophages, particularly MoMF, while neutrophils and liver lymphocytes were largely unaltered (Figure 6D and Figure S2: Immune cells in CCl_4_ induced liver fibrosis). Expression analyses of inflammation- (*Tnfα* and *Tgfβ*) and fibrosis- (*Acta2, Col1a1*, *Timp*, and *Ctgf*) related genes revealed minor changes upon GPR84 inhibition in the chronic CCl_4_ model (Figure 6F).

Taken together, GPR84 inhibition effectively inhibited myeloid cell infiltration upon liver injury induction in different experimental mouse models, consistently ameliorating liver inflammation and fibrosis upon chronic liver damage.

## 4. Discussion

Based on studies of insulin resistance, type 2 diabetes and obesity in rodents and humans, medium-chain fatty acids (MCFAs) are considered more favorable for glucose tolerance than long-chain fatty acids (LCFAs), when administered as dietary components [26,27,28,29]. Despite these beneficial effects of MCFAs on metabolism, they have more recently been linked to aggravated inflammatory processes. With respect to the pathogenesis of NAFLD and NASH, many potential drug targets have been identified over the last years, supporting the hypothesis that combination treatment targeting various signaling pathways at once might be more beneficial than single drug therapies [1,24,25]. For instance, promising targets modulate inflammatory, metabolic and fibrosis-related pathways, but the ideal combination of drugs has not been defined yet. MCFA, especially C9–C12 saturated fatty acids, are recognized by the surface receptor GPR84, which was first identified on granulocytes but is expressed on many innate immune cells [11,30]. GPR84 is upregulated on myeloid immune cells upon inflammatory stimuli, such as lipopolysaccharide and only to some extent on hepatocytes [22]. Interestingly, in a prior study, GPR84-deficient mice developed reduced hepatic steatosis compared to wildtype mice [13], supporting a metabolic disease-promoting role of MCFA-GPR84 signaling in the context of NAFLD. Moreover, GPR84-deficient mice were protected from organ fibrosis in experimental kidney injury [14]. We therefore hypothesized that pharmacological inhibition of GPR84 could be an interesting and novel target for the treatment of NASH.

In our study, we demonstrated that GPR84 is upregulated in human and mouse NAFLD and is associated with inflammation as well as fibrosis by histology. Human and mouse myeloid cells such as neutrophils and monocytes responded to MCFA as well as the GPR84 agonist embelin in chemotaxis assays, and two novel, distinct small molecule GPR84 antagonists were able to effectively inhibit this migratory response at nanomolar concentrations in vitro. These findings translated into a reduced macrophage accumulation in acute and chronic liver injury in vivo, upon treatment with GPR84 antagonists, and resulted in ameliorated steatohepatitis and fibrosis. Our study further indicated that the beneficial effects of GPR84 inhibition in chronic injury models in vivo are linked to the reduced myeloid cell infiltration of the liver. In particular, pharmacological inhibition of GPR84 had very little (if any) effects on hepatic steatosis, which was possibly explained by the fact that GPR84 expression on hepatocytes was low. Moreover, expression of GPR84 was not detectable on hepatic stellate cells (HSC), the key matrix-producing cell type responsible of liver fibrosis progression [9,31], supporting that the antifibrotic effects of GPR84 antagonists were related to their inhibition of myeloid cells (and not HSC directly).

Various lines of evidence support the therapeutic efficacy of inhibiting macrophage migration into injured liver [32]. Blocking macrophage accumulation in the liver was found to ameliorate disease manifestation in several preclinical models of chronic liver injury, mainly by interfering with the chemokine CCL2 and chemokine receptor CCR2 signaling [18,33,34,35,36]. Of particular interest to our current study, infiltrating monocyte-derived macrophages expressed high levels of fibrogenic mediators and were capable of activating hepatic stellate cells in vitro and in vivo [18,37,38], thereby supporting that blocking their infiltration into the injured liver would especially reduce hepatofibrogenesis. Early data from a clinical trial of the dual CCR2/CCR5 inhibitor cenicriviroc in patients with NASH indeed indicated an effective antifibrotic activity [39,40]. This strategy is currently being tested in a large phase 3 clinical trial [41]. However, the CCR2-CCL2 pathway is not specific to NAFLD/NASH, but is quite broadly involved in inflammatory monocyte recruitment [42]. In this context, targeting the MCFA receptor GPR84 is quite appealing, as it might represent a more NAFLD-specific pathway for recruiting inflammatory and fibrogenic macrophages to NASH livers.

GPR84 antagonists are currently under investigation in several disease areas, including pulmonary fibrosis and type 2 diabetes. For instance, PBI-4050 (3-pentylbenzeneacetic acid sodium salt), a synthetic analog of decanoic acid, is an orally active small molecule that acts as an agonist of GPR40 and as an antagonist of GPR84 [43]. This drug reduced experimental kidney as well as liver fibrosis and was found to block hepatic stellate cell activation via modulation of intracellular ATP levels and the LKB1/AMPK/mTOR pathway [14,43]. Moreover, GLPG1205, a nanomolar potency GPR84 antagonist, has demonstrated reduced lung fibrosis in two mouse models in a therapeutic setting [44]. Our study highlights the beneficial effects of small molecule GPR84 antagonists in experimental models of liver injury, steatohepatitis and fibrosis. Their high potency and specific activity in blocking MCFA/GPR84-mediated myeloid cell recruitment supports their further exploration as novel anti-inflammatory and anti-fibrotic agents in NASH.

## Figures and Tables

**Table 1:** Compound characteristics.

Assay Type	Cpd A	Cpd B
**h**GTPgS (IC_50_ nM) Hek293-**h**GPR84 cell membrane, DIM (EC_80_)	54 nM	11 nM
cAMP HTRF assay (IC_50_ nM) CHO-K1-**h**GPR84 cell membrane, Capric acid (EC_80_ = 61.7 µM)	7 nM	2 nM
cAMP HTRF assay (IC_50_ nM) CHO-K1-**m**GPR84 cell membrane, Capric acid (EC_80_ = 61.7 µM)	305 nM	34 nM
Human Neutrophil chemotaxis (IC_50_ nM), Embelin	11 nM	31 nM
Rat Neutrophil chemotaxis (IC_50_ nM), Embelin	111 nM	94 nM

**Table 2:** **Characteristics of human healthy controls and non-alcoholic fatty liver disease (NAFLD) patients.** Data on histology scoring are presented as median ± IQR. Abbreviations are: NAFLD, Non-alcoholic fatty liver disease; ALT, serum alanine transaminase; AST, serum aspartate transaminase; NAS, NAFLD Activity Score; n.a., not applicable.

	Healthy	NAFLD
n	5	19
Age, median (± IQR) years	41 (± 20)	59 (± 14.5)
ALT, median (± IQR) IU/l	n.a.	35 (± 38.5)
AST, median (± IQR) IU/l	n.a.	44.5 (± 43)
Liver Histology		
Kleiner score Steatosis	0 (± 0.0)	1.5 (± 1)
Kleiner score Ballooning	0 (± 0.0)	1 (± 1)
Kleiner score Inflammation	0 (± 0.0)	1.5 (± 1)
Kleiner NAS score	0 (± 0.0)	4.5 (± 2.4)
Kleiner Fibrosis stage	0 (± 0.75)	2 (± 1.5)

## Supplementary Materials

The following are available online at https://www.mdpi.com/2077-0383/9/4/1140/s1, Figure S1. Immune cells in MCD diet induced steatohepatitis and fibrosis. Figure S2. Immune cells in CCl4 induced liver fibrosis.

## References

[1] Friedman S.L., Neuschwander-Tetri B.A., Rinella M., Sanyal A.J. (2018). Mechanisms of NAFLD development and therapeutic strategies. Nat. Med..

[2] Younossi Z.M., Tacke F., Arrese M., Sharma B.C., Mostafa I., Bugianesi E., Wong V.W.-S., Yilmaz Y., George J., Fan J. (2019). Global Perspectives on Nonalcoholic Fatty Liver Disease and Nonalcoholic Steatohepatitis. Hepatology.

[3] Schattenberg J.M., Schuppan D. (2011). Nonalcoholic steatohepatitis. Curr. Opin. Lipidol..

[4] Hagström H., Nasr P., Ekstedt M., Hammar U., Stal P., Hultcrantz R., Kechagias S. (2017). Fibrosis stage but not NASH predicts mortality and time to development of severe liver disease in biopsy-proven NAFLD. J. Hepatol..

[5] Hundertmark J., Krenkel O., Tacke F. (2018). Adapted Immune Responses of Myeloid-Derived Cells in Fatty Liver Disease. Front. Immunol..

[6] Heymann F., Tacke F. (2016). Immunology in the liver—From homeostasis to disease. Nat. Rev. Gastroenterol. Hepatol..

[7] Krenkel O., Tacke F. (2017). Liver macrophages in tissue homeostasis and disease. Nat. Rev. Immunol..

[8] Mcgettigan B., Mcmahan R., Orlicky D., Burchill M., Danhorn T., Francis P., Cheng L.L., Golden-Mason L., Jakubzick C.V., Rosen H.R. (2019). Dietary Lipids Differentially Shape Nonalcoholic Steatohepatitis Progression and the Transcriptome of Kupffer Cells and Infiltrating Macrophages. Hepatology.

[9] Krenkel O., Hundertmark J., Abdallah A.T., Kohlhepp M., Puengel T., Roth T., Branco D.P.P., Mossanen J.C., Luedde T., Trautwein C. (2019). Myeloid cells in liver and bone marrow acquire a functionally distinct inflammatory phenotype during obesity-related steatohepatitis. Gut.

[10] Wang B., Li L., Fu J., Yu P., Gong D., Zeng C., Zeng Z. (2016). Effects of Long-Chain and Medium-Chain Fatty Acids on Apoptosis and Oxidative Stress in Human Liver Cells with Steatosis. J. Food Sci..

[11] Recio C., Lucy D., Purvis G.S.D., Iveson P., Zeboudj L., Iqbal A.J., Lin D., O’Callaghan C., Davison L., Griesbach E. (2018). Activation of the Immune-Metabolic Receptor GPR84 Enhances Inflammation and Phagocytosis in Macrophages. Front. Immunol..

[12] Husted A.S., Trauelsen M., Rudenko O., Hjorth S.A., Schwartz T.W. (2017). GPCR-Mediated Signaling of Metabolites. Cell Metab..

[13] Du Toit E., Browne L., Irving-Rodgers H., Massa H.M., Fozzard N., Jennings M.P., Peak I.R. (2017). Effect of GPR84 deletion on obesity and diabetes development in mice fed long chain or medium chain fatty acid rich diets. Eur. J. Nutr..

[14] Gagnon L., LeDuc M., Thibodeau J.-F., Zhang M.-Z., Grouix B., Sarra-Bournet F., Gagnon W., Hince K., Tremblay M., Geerts L. (2018). A Newly Discovered Antifibrotic Pathway Regulated by Two Fatty Acid Receptors. Am. J. Pathol..

[15] Guldiken N., Ensari G.K., Lahiri P., Couchy G., Preisinger C., Liedtke C., Zimmermann H.W., Ziol M., Boor P., Zucman-Rossi J. (2016). Keratin 23 is a stress-inducible marker of mouse and human ductular reaction in liver disease. J. Hepatol..

[16] Kleiner D.E., Brunt E.M., Van Natta M., Behling C., Contos M.J., Cummings O.W., Ferrell L.D., Liu Y.-C., Torbenson M.S., Unalp-Arida A. (2005). Design and validation of a histological scoring system for nonalcoholic fatty liver disease. Hepatology.

[17] Weiskirchen R., Loomba R., Lawitz E., Mantry P.S., Jayakumar S., Caldwell S.H., Arnold H., Diehl A.M., Djedjos C.S., Han L. (2018). Faculty of 1000 evaluation for The ASK1 inhibitor selonsertib in patients with nonalcoholic steatohepatitis: A randomized, phase 2 trial. F1000 - Post-Publ. Peer Rev. Biomed. Lit..

[18] Krenkel O., Puengel T., Govaere O., Abdallah A.T., Mossanen J.C., Kohlhepp M., Liepelt A., Lefebvre E., Luedde T., Hellerbrand C. (2018). Therapeutic inhibition of inflammatory monocyte recruitment reduces steatohepatitis and liver fibrosis. Hepatology.

[19] Puengel T., Krenkel O., Kohlhepp M., Lefebvre E., Luedde T., Trautwein C., Tacke F. (2017). Differential impact of the dual CCR2/CCR5 inhibitor cenicriviroc on migration of monocyte and lymphocyte subsets in acute liver injury. PLoS ONE.

[20] Gaidarov I., Anthony T., Gatlin J., Chen X., Mills D., Solomon M., Han S., Semple G., Unett D.J. (2018). Embelin and its derivatives unravel the signaling, proinflammatory and antiatherogenic properties of GPR84 receptor. Pharmacol. Res..

[21] Wang J., Wu X., Simonavicius N., Tian H., Ling L. (2006). Medium-chain Fatty Acids as Ligands for Orphan G Protein-coupled Receptor GPR84. J. Boil. Chem..

[22] Suzuki M., Takaishi S., Nagasaki M., Onozawa Y., Iino I., Maeda H., Komai T., Oda T. (2013). Medium-chain Fatty Acid-sensing Receptor, GPR84, Is a Proinflammatory Receptor. J. Boil. Chem..

[23] Liedtke C., Luedde T., Sauerbruch T., Scholten D., Streetz K., Tacke F., Tolba R.H., Trautwein C., Trebicka J., Weiskirchen R. (2013). Experimental liver fibrosis research: Update on animal models, legal issues and translational aspects. Fibrogenesis Tissue Repair.

[24] Reimer K.C., Wree A., Roderburg C., Tacke F. (2019). New drugs for NAFLD: Lessons from basic models to the clinic. Hepatol. Int..

[25] Tacke F., Weiskirchen R. (2018). An update on the recent advances in antifibrotic therapy. Expert Rev. Gastroenterol. Hepatol..

[26] Shinohara H., Ogawa A., Kasai M., Aoyama T. (2005). Effect of Randomly Interesterified Triacylglycerols Containing Medium- and Long-Chain Fatty Acids on Energy Expenditure and Hepatic Fatty Acid Metabolism in Rats. Biosci. Biotechnol. Biochem..

[27] Turner N., Hariharan K., TidAng J., Frangioudakis G., Beale S.M., Wright L.E., Zeng X., Leslie S.J., Li J.-Y., Kraegen E.W. (2009). Enhancement of Muscle Mitochondrial Oxidative Capacity and Alterations in Insulin Action Are Lipid Species Dependent. Diabetes.

[28] St-Onge M.-P., Jones P.J.H. (2002). Physiological Effects of Medium-Chain Triglycerides: Potential Agents in the Prevention of Obesity. J. Nutr..

[29] St-Onge M.-P., Ross R., Parsons W.D., Jones P.J.H. (2003). Medium-Chain Triglycerides Increase Energy Expenditure and Decrease Adiposity in Overweight Men. Obes. Res..

[30] Yousefi S., Cooper P.R., Potter S.L., Mueck B., Jarai G. (2001). Cloning and expression analysis of a novel G-protein-coupled receptor selectively expressed on granulocytes. J. Leukoc. Boil..

[31] Krenkel O., Hundertmark J., Ritz T.P., Weiskirchen R., Tacke F. (2019). Single Cell RNA Sequencing Identifies Subsets of Hepatic Stellate Cells and Myofibroblasts in Liver Fibrosis. Cells.

[32] Tacke F. (2017). Targeting hepatic macrophages to treat liver diseases. J. Hepatol..

[33] Guicciardi M.E., Trussoni C.E., Krishnan A., Bronk S.F., Pisarello M.J.L., O’Hara S.P., Splinter P.L., Gao Y., Vig P., Revzin A. (2018). Macrophages contribute to the pathogenesis of sclerosing cholangitis in mice. J. Hepatol..

[34] Ambade A., Lowe P., Kodys K., Catalano D., Gyongyosi B., Cho Y., Iracheta-Vellve A., Adejumo A., Saha B., Calenda C. (2019). Pharmacological Inhibition of CCR2/5 Signaling Prevents and Reverses Alcohol-Induced Liver Damage, Steatosis, and Inflammation in Mice. Hepatology.

[35] Baeck C., Wehr A., Karlmark K.R., Heymann F., Vucur M., Gassler N., Huss S., Klussmann S., Eulberg D., Luedde T. (2011). Pharmacological inhibition of the chemokine CCL2 (MCP-1) diminishes liver macrophage infiltration and steatohepatitis in chronic hepatic injury. Gut.

[36] Bartneck M., Schrammen P.L., Möckel D., Govaere O., Liepelt A., Krenkel O., Ergen C., McCain M.V., Eulberg D., Luedde T. (2019). The CCR2+ Macrophage Subset Promotes Pathogenic Angiogenesis for Tumor Vascularization in Fibrotic Livers. Cell. Mol. Gastroenterol. Hepatol..

[37] Karlmark K.R., Weiskirchen R., Zimmermann H.W., Gassler N., Ginhoux F., Weber C., Merad M., Luedde T., Trautwein C., Tacke F. (2009). Hepatic recruitment of the inflammatory Gr1+monocyte subset upon liver injury promotes hepatic fibrosis. Hepatology.

[38] Pradère J.-P., Kluwe J., De Minicis S., Jiao J.-J., Gwak G.-Y., Dapito D.H., Jang M.-K., Guenther N.D., Mederacke I., Friedman R. (2013). Hepatic macrophages but not dendritic cells contribute to liver fibrosis by promoting the survival of activated hepatic stellate cells in mice. Hepatology.

[39] Friedman S.L., Ratziu V., Harrison S.A., Abdelmalek M.F., Aithal G.P., Caballeria J., Francque S., Farrell G., Kowdley K.V., Craxi A. (2018). A randomized, placebo-controlled trial of cenicriviroc for treatment of nonalcoholic steatohepatitis with fibrosis. Hepatology.

[40] Ratziu V., Sanyal A., Harrison S.A., Wong V.W., Francque S., Goodman Z., Aithal G.P., Kowdley K.V., Seyedkazemi S., Fischer L. (2020). Cenicriviroc Treatment for Adults with Nonalcoholic Steatohepatitis and Fibrosis: Final Analysis of the Phase 2b CENTAUR Study. Hepatology.

[41] Anstee Q.M., Neuschwander-Tetri B.A., Wong V.W.-S., Abdelmalek M.F., Younossi Z., Alkhouri N., Yuan J., Pecoraro M.L., Seyedkazemi S., Fischer L. (2019). Cenicriviroc (CVC) for the Treatment of Liver Fibrosis in Adults with Nonalcoholic Steatohepatitis (NASH): AURORA Phase 3 Study Design. Am. J. Gastroenterol..

[42] Tacke F. (2018). Cenicriviroc for the treatment of non-alcoholic steatohepatitis and liver fibrosis. Expert Opin. Investig. Drugs.

[43] Grouix B., Sarra-Bournet F., Leduc M., Simard J.C., Hince K., Geerts L., Blais A., Gervais L., Laverdure A., Felton A. (2018). PBI-4050 Reduces Stellate Cell Activation and Liver Fibrosis through Modulation of Intracellular ATP Levels and the Liver Kinase B1/AMP-Activated Protein Kinase/Mammalian Target of Rapamycin Pathway. J. Pharmacol. Exp. Ther..

[44] Saniere L., Marsais F., Jagerschmidt C., Meurisse S., Cuzic S., Shoji K., Clement-Lacroix P., Van Osselaer N., De Vos S. (2019). Characterization of GLPG1205 in Mouse Fibrosis Models: A Potent and Selective Antagonist of GPR84 for Treatment of Idiopathic Pulmonary Fibrosis. Am. J. Respir. Crit. Care Med..

