# Supplementary material for: The Medium-Chain Fatty Acid Receptor GPR84 Mediates Myeloid Cell Infiltration Promoting Steatohepatitis and Fibrosis
Source: J Clin Med. 2020 Apr 16;9(4):1140. doi: 10.3390/jcm9041140 (PMC7231190; doi:10.3390/jcm9041140)
Supplement: Supplementary file 1 [file jcm-09-01140-s001.pdf]

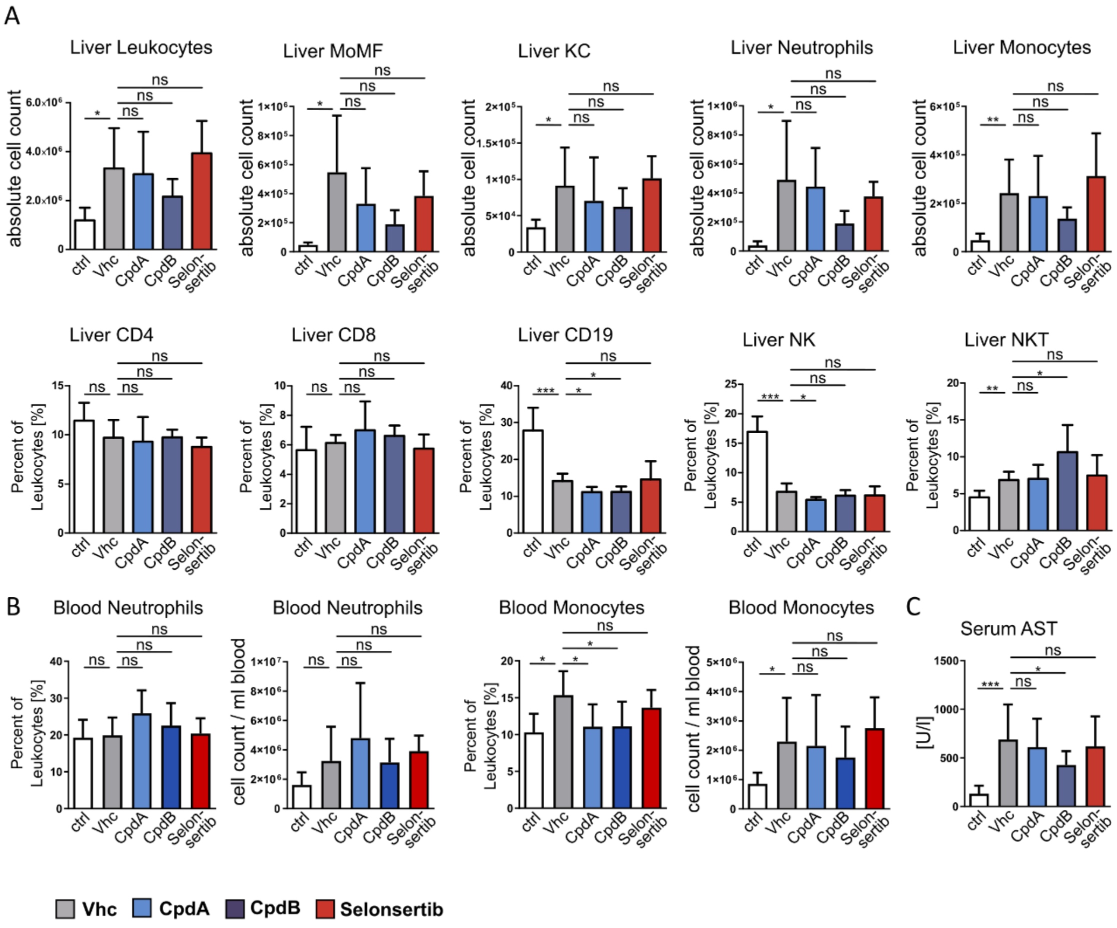

Figure S1. Immune cells in MCD diet induced steatohepatitis and fibrosis. (A) Quantification of liver myeloid and lymphoid immune cell populations by FACS analysis. (B) Blood neutrophils and monocytes quantified by FACS analysis in absolute numbers and as percent of leukocytes. (C) Aspartate aminotransferase (AST) serum levels. Data are presented as mean ± SD based on n≥8 per group. *p<0.05, **p<0.01, ***p<0.001 (one-way ANOVA). Abbreviations are: CpdA/B, compound A/B.

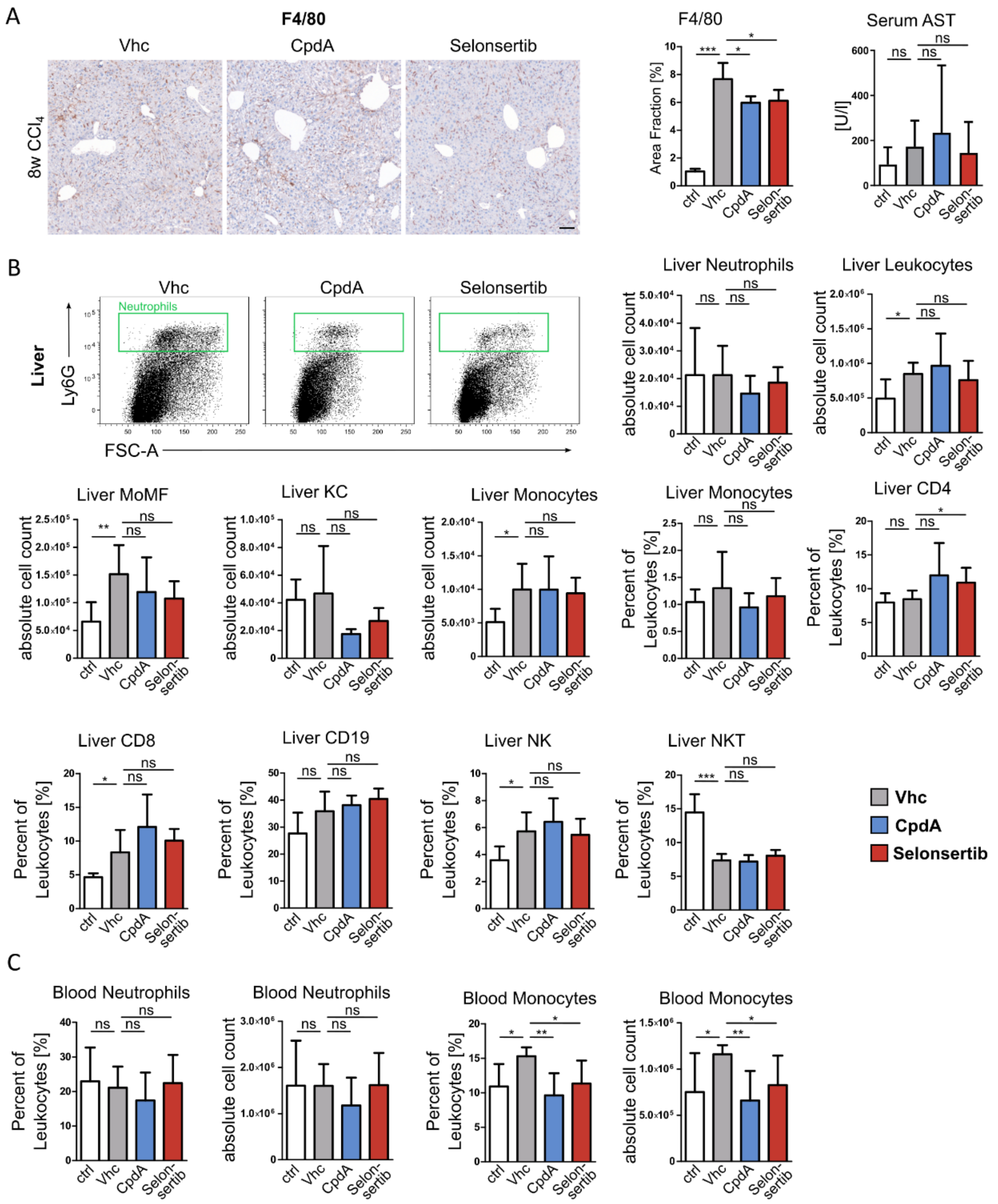

Figure S2. Immune cells in CCl_4 induced liver fibrosis. (A) Representative F4/80 immunohistochemistry staining, corresponding quantification of F4/80 positive areas and serum aspartate aminotransferase (AST) levels. (B) Representative FACS plots showing Ly6G+ liver neutrophils. Quantification of hepatic myeloid and lymphoid immune cell populations on absolute numbers and percent of liver leukocytes. (C) FACS analysis of blood neutrophils and monocytes. Data are presented as mean ± SD based on n≥8 per group. *p<0.05, **p<0.01, ***p<0.001 (one-way ANOVA). Abbreviations are: CpdA, compound A.
